# Annotations

**Published:** 1908-06-27

**Authors:** 


### ANNOTATIONS.

### What is Margarine?

Some time ago one of the inspectors of food and drugs bought at a shop in Derby a material which was made up to look like butter, but was called "nutter," and was composed of vegetable fats. The inspector thought himself justified in prosecuting the seller under the Margarine Act of 1887, on the ground that the substance was margarine within the meaning of the Act, and should have been so labelled, whereas it was labelled "nut-cream butter," and the name popularly shortened into "nutter." The seller contended that this nut butter was unknown at the time of the passing of the Act, and that what is known as margarine is a compound of animal fat, and on these grounds justified his action. Not being made of milk and cream, he could not call the substance butter; not being made of animal fat, it was not margarine; and "nutter" seemed to him a proper definition for it. This view was upheld by the justices, but when on appeal the case was taken into the High Court, it was contended on behalf of the inspector that "margarine" meant "all substances, whether compounds or otherwise, prepared in imitation of butter, and whether mixed with butter or not," and this view was taken by the Court as a correct interpretation of the Act. The decision raises a point that is of considerable interest now that vegetarianism is becoming popular. If both animal and vegetable substances are to be labelled "margarine" it is not possible for the vegetarian to know whether or not he is getting what he wants. On the other hand, it is possible that vegetable butter will be palmed off as "margarine" on people who want animal fat. Margarine is used in many institutions, and it is doubtful if either the authorities or the inmates of these want nut butter. Either "nutter" must be recognised as a substance by itself, or else the sellers of margarine should be so instructed that they will be able to tell intending purchasers of what their wares are composed—and this would not be easy. But institutions should meanwhile demand an analysis of the margarine tendered them.

### The Sleeping Canopy.

There are often practical difficulties which militate against the adoption of tents or other outdoor sleeping arrangements for tuberculous patients whom by force of circumstances their medical attendants are compelled to treat at home. To overcome these an American physician, Dr. Charles Denison, has devised a substitute for the outdoor sleeping tent which he calls a "sleeping canopy." The principle is simple: the bed is brought close up to the window of the sleeping apartment, either sideways or head on, and by means of curtains fastened around the outer rim of the window casing, supported by a light framework, and long enough to reach the floor all round the bed, a little alcove is made which is entirely shut off from the room, and is, when the window is open, to all intents an outdoor shelter. Several advantages are claimed for this bed canopy: thus the window may be wholly, or but partly open, and the invalid may experiment with more and more air; when he arises in the morning or during the night, he comes at once into a warm room; no draughts can circulate, and if a strong wind comes up, the air supply can be limited by means of the window. It is said that the reluctant can be thus gradually coaxed into the habit of outdoor sleeping. Dr. Denison believes that there is no more danger of catching cold by the use of his invention than there is for the backwoodsman who habitually camps out in all weathers; it is all a question of habit, he says, and if sufficient wraps are provided the most susceptible invalid may sleep in the canopy with window wide open in any weather. So far, most physicians will agree cordially, and also in offering a hearty welcome to his indoor tent as an addition to the accessories for treating tuberculosis in cities. He proceeds to emphasise the part played by cramped air-supply in the production of tuberculosis, and would explain "tuberculous houses" on this ground, rather than on that of germ concentration and opportunity for infection thereby. There is probably a good deal to be said for his view; but in neglecting altogether the germ question, he may be as much in error as are those who neglect that of air supply. There seems no sufficient reason why both factors may not enter into the ætiology of tuberculous infection.

### The Motor 'Bus Nuisance.

For some time past there has been a growing conviction among many of the inhabitants of London that something should and could be done to abate the dangers and annoyance due to the heavy motor traffic of the streets. The excessive noise, size, and speed of motor omnibuses and other large power-drawn vehicles, and the abominable fumes which they constantly emit, have rendered many of the main thoroughfares of London quite intolerable. Private irritation has now at last found a means of expressing itself in public and of making representations to the proper quarters. At an influential meeting held on June 22 at the Mansion House, under the direction of the Lord Mayor, the subject of offensive and dangerous motor traffic was discussed, and resolutions were passed urging the necessity for legislative measures to be passed for the abatement of this increasing nuisance, and recommending that a Central Traffic Board should be formed for the control and regulation of street traffic in London. To give effect to these resolutions, a deputation was appointed to wait upon the Home Secretary, the President of the Local Government Board, and the President of the Board of Trade. A letter, which was read from Sir Dyce Duckworth, contained a trenchant criticism of the present unsatisfactory state of affairs. As he very wisely says, there is no objection to be made to motor traffic as such, but against the manner in which it is allowed to be conducted. The motor 'bus traffic has, indeed, become "an intolerable intrusion upon the safety and comfort of the pedestrians and travellers on the line of its routes," and some official means of controlling the use and construction of motor vehicles, especially of the more powerful type is urgently needed.

